# Optimization of Loop-Mediated Isothermal Amplification for Avian Influenza Detection

**DOI:** 10.3390/ani15202983

**Published:** 2025-10-15

**Authors:** Anastasia Glazunova, Timofey Sevskikh, Dmitry Kudryashov, Irina Sindryakova, Olga Kolbasova, Maria Erokhina, Andrey Mukhin, Denis Kolbasov, Ilya Titov

**Affiliations:** 1 Federal Research Center for Virology and Microbiology, Academician Bakoulov Street, bldg. 1, Petushki Area, 601125 Volginsky, Vladimir Oblast, Russia; dima_kudryashov@mail.ru (D.K.); sindryakova.irina@yandex.ru (I.S.); olgakolbasova@gmail.com (O.K.); kolbasovdenis@gmail.com (D.K.); titoffia@yandex.ru (I.T.); 2 Russian Academy of Sciences, Biological Station Rybachy of the Zoological Institute, 32 Pobedy Street, Zelenogradsky Municipal Okrug, 238535 Rybachy, Kaliningrad Oblast, Russia; erokhina.marija@yandex.ru (M.E.); a.l.mukhin@gmail.com (A.M.)

**Keywords:** avian influenza virus (AIV), molecular diagnostics, RT-PCR, colorimetric visualization, RT-LAMP, analytical sensitivity and specificity

## Abstract

Avian influenza viruses (AIV) cause severe economic losses in poultry production and pose zoonotic threats, necessitating rapid, field-deployable diagnostics. While real-time PCR is the gold standard, its use is limited in resource-constrained settings. This study aimed to develop and validate optimized loop-mediated isothermal amplification (LAMP) protocols for AIV detection directly at sample collection sites. We optimized Real-Time RT-LAMP and colorimetric LAMP assays targeting the conserved M gene, using primers described in the literature. Analytical sensitivity was assessed with a plasmid standard (10^6^–10^0^ copies/μL); specificity was evaluated against 27 AIV strains (H1–H12) and heterologous avian viruses (Newcastle disease, infectious bronchitis, Gumboro, and laryngotracheitis viruses). Reverse transcription was integrated into the LAMP reaction. Real-Time LAMP with SYBR Green achieved 100% analytical sensitivity (95% CI: 80–100; detection limit: Ct = 38), while colorimetric LAMP (cresol red, malachite green, calcein) detected 10^2^ plasmid copies (Ct = 32) with 91.67% sensitivity (95% CI: 76.1–100). No cross-reactivity occurred. These optimized LAMP protocols offer sensitivity and specificity comparable to PCR, require minimal equipment, and enable rapid AIV screening, significantly enhancing early detection and epidemiological surveillance in field conditions.

## Simple Summary

Avian influenza viruses (AIV) pose a significant threat to poultry health and food security, necessitating rapid and reliable diagnostic methods. This study presents optimized protocols for detecting AIV using Real-Time RT-LAMP and colorimetric LAMP with multiple visual indicators (cresol red, malachite green, calcein). Validation demonstrated that Real-Time LAMP with SYBR Green achieved performance comparable to real-time PCR, with a detection limit corresponding to Ct = 38 and 100% analytical sensitivity (95% CI: 80–100). The colorimetric LAMP format showed a sensitivity limit of 10^2^ plasmid copies (Ct = 32 in RT-PCR) with 91.67% analytical sensitivity (95% CI: 76.1–100). Specificity testing confirmed amplification exclusively in AIV samples (subtypes H1–H12), with no cross-reactivity to other avian viruses or negative controls. Although field monitoring during the study period revealed no natural AIV infections, comprehensive parallel testing with real-time PCR established 100% concordance across all field samples (*n* = 69), confirming the method’s diagnostic reliability. The optimized LAMP protocols provide a robust, rapid detection system suitable for resource-limited settings, enabling early outbreak identification and enhancing AIV surveillance capabilities without requiring complex instrumentation.

## 1. Introduction

Avian influenza viruses (AIV), particularly highly pathogenic avian influenza (HPAI) strains such as H5N1, pose a severe threat to global poultry production, causing massive economic losses and disrupting food supply chains. Outbreaks have led to the culling of hundreds of millions of birds worldwide, with significant impacts on commercial farms and small-scale producers alike. For example, the 2014–2015 HPAI outbreak in the United States resulted in losses exceeding $1.6 billion [1,2,3,4,5]. Beyond animal health, there is growing concern about zoonotic transmission, highlighted by recent cases of human H5N1 infections linked to contact with infected animals, including cattle and poultry. For instance, in late January 2025, a human case of HPAI subtype H5N1 was reported on a farm in the UK following prolonged contact with infected birds [6]. This highlights the importance of continuous monitoring and scientific research in this field [2].

Influenza viruses, members of the Orthomyxoviridae family, are classified into four genera (A, B, C, and D), with influenza A viruses exhibiting the greatest genetic and antigenic diversity, broad host tropism, and epidemic potential due to their segmented genome (eight single-stranded negative-sense RNA segments) that enables antigenic drift and reassortment; in contrast, influenza B viruses (divided into Victoria and Yamagata lineages) primarily cause seasonal human flu, influenza C viruses (with only seven genome segments) cause mild respiratory illness in humans and pigs, and influenza D virus—first isolated from swine in 2011—shows no cross-reactivity with type C despite ~50% amino acid identity [7,8,9,10,11,12]. The primary natural reservoirs of AIV are wild migratory birds, particularly species within the orders *Anseriformes* (ducks, geese, swans) and *Charadriiformes* (gulls, terns) [13,14,15,16,17,18]. These birds often carry the virus asymptomatically and can disseminate it over vast distances along migratory flyways, serving as a key driver of intercontinental spread [13]. The ongoing circulation of HPAI H5N1 clade 2.3.4.4b among wild bird populations since 2020 has led to unprecedented geographic expansion, affecting over 3000 outbreaks globally in 2024 alone, with hotspots in Europe and the Americas [19]. Notably, the virus has increasingly been detected in mammals, including domestic cats, dairy cattle, and marine species, indicating an expanding host range and heightened pandemic risk [20,21]. Rapid and accurate diagnosis is critical for effective disease control. Real-time RT-PCR is considered the gold standard for AIV detection due to its high sensitivity and specificity [22]. However, its widespread use is limited by high equipment costs, the need for specialized laboratory infrastructure, trained personnel, and long turnaround times [23,24,25]. These constraints are especially problematic in resource-limited settings and during field surveillance, where timely results are essential.

To address these limitations, loop-mediated isothermal amplification (LAMP) has emerged as a promising alternative. LAMP offers rapid results (30–60 min), high sensitivity, and minimal equipment requirements, making it suitable for point-of-care and field applications [26,27]. It has been successfully used for detecting various pathogens [28,29,30], including SARS-CoV-2, dengue virus, and African swine fever virus [31,32,33,34,35]. Commercial tests based on isothermal amplification already exist, using specialized equipment, including systems for isothermal fluorescent -LAMP from various manufacturers [36,37,38]. Despite its advantages, challenges remain, including optimization of primers, selection of reliable visual indicators, and validation across diverse field conditions.

This study optimized a cost-effective and efficient LAMP assay for the detection of AIV using various reagents and detection methods. The developed protocols RT-LAMP and colorimetric formats with different indicators (SYBR Green, cresol red, malachite green, calcein). For each protocol, analytical sensitivity and specificity were determined. The proposed modifications were shown to enhance test system reliability, reduce the frequency of false-positive results, and expand application capabilities in field conditions. The obtained results contribute to overcoming existing barriers to the implementation of the LAMP method in veterinary diagnostic practice, which is particularly relevant given the critical importance of avian influenza surveillance and the potential of LAMP for both laboratory and field applications.

## 2. Materials and Methods

### 2.1. The Material Under Study

The study was conducted at the Federal Research Center for Virology and Microbiology (FRCVM) in the Laboratory of Molecular Virology. All experimental work was carried out in compliance with sanitary and epidemiological requirements established by SanPiN 3.3686-21 [39]. Procedures were performed in two isolated rooms (zones) according to methodological guidelines MU 1.3.2569-09 [40].

The method’s analytical sensitivity was assessed using serial decimal dilutions (10^6^–10^0^ copies/μL) of a recombinant plasmid carrying a 206 bp fragment of AIV M gene.

To evaluate the specificity of the method, reactions were set up using samples from the FRCVM research collection, including AIV and heterologous viruses of birds affecting similar hosts (Newcastle disease virus, avian infectious bronchitis virus, Gumboro disease virus, and avian infectious laryngotracheitis virus). The list of tested samples is provided in Table 1.

To assess the analytical sensitivity and analytical specificity of optimized LAMP methods, samples of various strains of AIV and heterologous avian viruses were used. The analytical sensitivity was also evaluated for a plasmid containing an insert of the M gene fragment of the AIV virus. Statistical analysis was performed using R software (version 4.3.1), in which a table was created with the following indicators: TP (true positives, i.e., the number of samples correctly identified as positive by the LAMP method and confirmed as positive by real-time PCR), FP (false positives, i.e., the number of samples incorrectly identified as positive by the LAMP method but confirmed as negative by PCR), TN (true negatives, i.e., the number of samples correctly identified as negative by the LAMP method and confirmed as negative by PCR), FN (false negatives, i.e., the number of samples incorrectly identified as negative by the LAMP method but confirmed as positive by PCR). To calculate the 95% confidence intervals for analytical sensitivity and analytical specificity, we used the «binom» package (https://cran.r-project.org/web/packages/binom/index.html accessed on 23 May 2025). Kappa coefficient was assessed using the «psych» package with the «kappa» function (https://cran.r-project.org/web/packages/psych/vignettes/ accessed on 23 May 2025), enabling statistical evaluation of inter-method agreement. The kappa coefficient was interpreted according to conventional criteria: values ranging from 0.81 to 1.00 indicate almost perfect agreement; 0.61 to 0.80, substantial agreement; 0.41 to 0.60, moderate agreement; 0.21 to 0.40, fair agreement; and 0.00 to 0.20, slight agreement. Negative values indicate no agreement between the compared methods.

### 2.2. Molecular Cloning of the Plasmid

A 206 bp fragment of the M gene from the AIV strain A/rook/Russia/A_rook_Nizhniy_Novgorod_2_17/2017(H5N8) was amplified by PCR using LAMP outer primers F3 and B3 specific to the M gene, of design by Golabi et al. (2021) [41], followed by cloning into the pAL2T plasmid vector (Eurogen, Moscow, Russia). For cloning, the commercial Quick-TA kit (Eurogen, Russia) was used. Ligation was carried out using T4 ligase (Eurogen, Russia). Competent cells (strain XL-1 *E. coli*, Eurogen, Russia) were used for transformation. Plasmid DNA extraction was performed using the Plasmid Miniprep kit (Eurogen, Russia). All experimental procedures were strictly performed according to the manufacturer’s recommendations and instructions.

Quantitative assessment of plasmid concentration was performed using the ND-100 microvolume spectrophotometer (Miulab, Zhejiang, China). A series of tenfold dilutions of the plasmid was prepared for further testing and validation of the AIV-LAMP method, starting from an initial concentration of 10^10^ plasmid copies/µL down to 4.9 plasmid copy/µL.

### 2.3. Optimization of the LAMP Reaction

Optimization of the LAMP reaction for detecting the AIV genome was performed in two formats: fluorescent detection (SYBR Green) and colorimetric indicators. For the colorimetric method, the following indicators were used: pH-dependent cresol red, working in the pH range of 7.2–8.8; malachite green, which changes color during amplification; colorimetric fluorescent indicator calcein.

To optimize and accelerate the reaction, the reverse transcription and amplification stages were combined in a single tube for analyzing samples from the research collection. Primers designed by Golabi M. et al., targeting the M gene of type A influenza virus, were used for all experiments, including testing both plasmids and samples [41].

The reaction mixture for LAMP with SYBR Green (Real-Time LAMP) included three pairs of published primers [41]: FIP and BIP (0.8 µM), LoopF and LoopB (0.4 µM), F3 and B3 (0.2 µM), as well as 5X LAMP buffer (GEN TERRA, Moscow Russia), 40 U GentaBst DNA polymerase (GEN TERRA, Russia), 10 mM dNTP (diaGene, Moscow Russia), 50 U MMIv reverse transcriptase (GEN TERRA, Russia), 12.5% SYBR Green (Eurogen, Russia), and nuclease-free water. The total reaction volume was 25 µL (Table S1). Amplification was performed on the AXXIN T8 isothermal amplifier (Australia) at a constant reaction temperature of 58 °C for 30 min. Data was recorded based on changes in amplification curves.

The reaction mixture for colorimetric LAMP reactions using Malachite Green and calcein followed a protocol analogous to the SYBR Green fluorescent method. For Malachite Green, a 0.3% solution was used, while for calcein, a 2.5 mM solution was used in the presence of 25 mM MnCl_2_ (Tables S2 and S3). Amplification was carried out in the solid-state thermoblock “Gnom” (DNA-Technology, Moscow, Russia) at constant temperatures of 61 °C and 63 °C, respectively, for each dye. Results were evaluated based on color changes in the reaction mixture: for Malachite Green: blue = positive, colorless = negative; for calcein: presence of fluorescence = positive, absence of fluorescence = negative; color change (yellow-green = positive, light brown = negative). Calcein fluorescence was detected using a transilluminator (Vilber Lourmat, Collégien, France) with a wavelength of 254 nm.

For cresol red-based colorimetric LAMP an adapted protocol developed by Felipe Navarro Martínez et al. [42] was used. Modifications included the replacement of the indicator and its direct addition to the reaction buffer, while maintaining the concentrations of other components as specified in the original protocol.

The reaction mixture consisted of published primers [41]: FIP and BIP (0.8 µM), LoopF and LoopB (0.4 µM), F3 and B3 (0.2 µM), a buffer containing dNTPs, (NH_4_)_2_SO_4_, MgSO_4_, KCl, Tween 20, and cresol red as the indicator, 40 U GentaBst DNA polymerase (GEN TERRA, Russia), 50 U MMIv reverse transcriptase (GEN TERRA, Russia), and nuclease-free water (Tables S4 and S5). Isothermal amplification was performed in the “Gnom” thermoblock (DNA-Technology, Russia) at 60 °C for 30 min. Results were evaluated based on color changes in the reaction mixture (yellow = positive, red = negative), assessed visually without specialized equipment.

### 2.4. Verification of LAMP Results by Agarose Gel Electrophoresis

Reaction products were analyzed by agarose gel electrophoresis in a 1% gel containing 0.001% ethidium bromide intercalating dye. Electrophoresis was performed in TAE buffer at a voltage of 120 mA for 40–60 min. Visualization of results was carried out using the GelDocXR system (Bio-Rad Laboratories, Hercules, CA, USA), registering specific bands in the tracks of the tested samples compared to a molecular weight marker.

### 2.5. Detection of AIV by Real-Time PCR

For evaluating the sensitivity of samples from the research collection, nucleic acids were extracted using a commercial kit based on magnetic separation technology (Magno-prime vet, Moscow, Russia). Synthesis of complementary DNA (cDNA) was performed using a commercial reverse transcription kit (REVERTA-L, Moscow, Russia).

For real-time PCR detection of AIV, synthesized cDNA and a series of tenfold plasmid dilutions were used as samples.

Amplification protocols were adapted and validated according to recommendations from the FLI [43]. During PCR, primers and probes recommended by FLI, 5X qPCRmix-HS ready-to-use buffer (Eurogen, Russia), and nuclease-free water were employed. Amplification was performed on the CFX96 real-time PCR detection system (Bio-Rad, USA) with signal registration in the FAM channel. The thermal cycling profile (without reverse transcription stage) included the following steps: Taq polymerase activation at 95 °C for 1 min, denaturation at 95 °C for 15 s, annealing at 56 °C for 20 s, extension at 72 °C for 30 s. The total number of amplification cycles was 45.

### 2.6. Field Verification of Optimized LAMP Protocols

Verification of optimized LAMP protocols included evaluation of amplification specificity to exclude false-positive reactions. The study was conducted in mid-April 2025 during field monitoring for avian influenza in an epizootically significant region of the Russian Federation—the Kaliningrad Oblast (Curonian Spit). This territory serves as a key migration corridor with a high risk of AIV introduction from affected neighboring countries (Poland and Lithuania). To assess amplification specificity, a variety of samples from different biological materials and various bird species were selected and analyzed. A total of 69 samples were analyzed, including: oropharyngeal and cloacal swabs from 28 bird species of the order *Passeriformes* (*n* = 56; 28 of each type) and fecal samples from 13 waterfowl specimens (*Larus argentatus*, *Chroicocephalus ridibundus*, *Ardea cinerea*, *Anas platyrhynchos*, *Phalacrocorax carbo*, *Jack snipe*, and *Cygnus olor*). Optimized LAMP assays were performed according to the developed protocols using extracted nucleic acids as amplification templates. All samples were simultaneously tested by real-time PCR.

## 3. Results

### 3.1. Experimental Results and Data Analysis of PCR Setup

**Figure 1:**
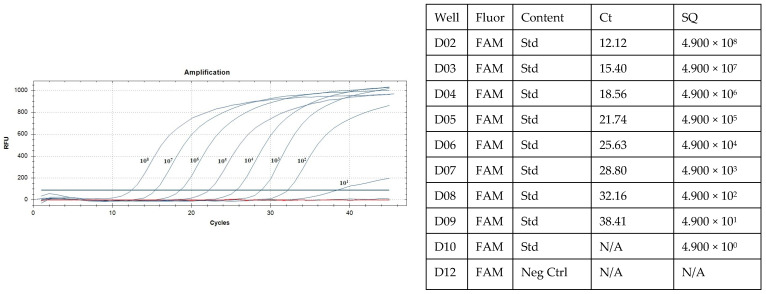
Determination of real-time PCR sensitivity for detecting fragments of the AIV M gene. Numbers indicate the degree of plasmid dilution concentration (blue curves) and negative controls (red curve), N/A—not applicable.

The sensitivity analysis using real-time PCR with various plasmid concentrations allowed us to determine the detection threshold Ct (the threshold cycle indicates the number of PCR amplification cycles needed to attain the predetermined fluorescence threshold level), i.e., the minimal amount of DNA that could be reliably detected and quantified. In the usual PCR reaction, the plasmid was detectable in concentrations ranging from 10^8^ to 10^1^ (Figure 1).

To assess the diagnostic specificity of the method and conduct a comparative analysis with the developed LAMP protocols, parallel testing was performed using real-time PCR. The study utilized a panel of samples comprising reference strains of avian influenza virus (AIV) subtypes H1-H12 (*n* = 27), as well as viruses circulating among birds: Newcastle disease virus (2 strains), avian infectious bronchitis virus (2 strains), infectious bursal disease virus (1 strain), and avian infectious laryngotracheitis virus (2 strains) from the institute’s research collection. All samples had been previously characterized and represented different bird species, geographical isolates, and temporal periods of isolation.

The real-time PCR method demonstrated 100% analytical specificity, correctly identifying all 27 AIV strains and showing no cross-reactions with heterologous viruses. Detailed characteristics of the tested samples and real-time PCR results are presented in Table 1.

### 3.2. Experimental Results and Data Analysis of Optimized LAMP Reactions

**Figure 2:**
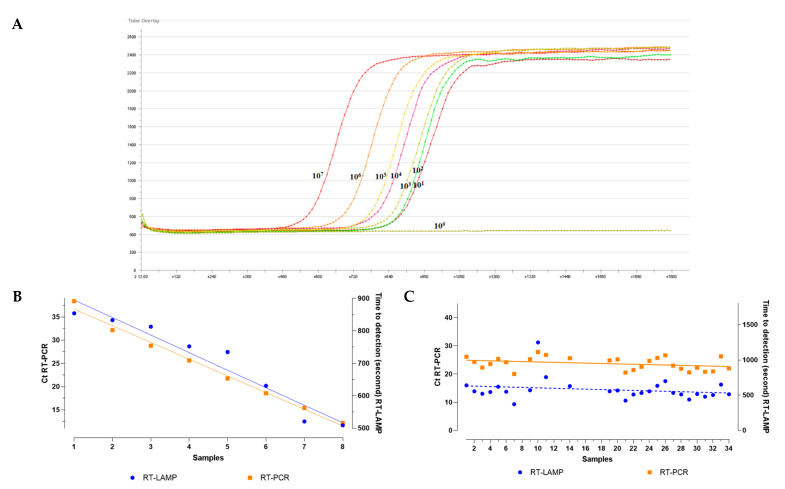
Optimized Real-Time LAMP protocol with SYBR Green (12.5X) fluorescent indicator and its sensitivity comparison with real-time PCR. (**A**) Amplification kinetics. Amplification curves (in seconds) are shown for testing a plasmid containing a 206 bp insert of the AIV M gene fragment in tenfold serial dilutions. Analysis was performed using SYBR Green (12.5X) fluorescent indicator on the AXXIN T8 isothermal amplifier (Australia). (**B**) Sensitivity comparison of methods. Results of detection limit comparison between the optimized Real-Time LAMP protocol and real-time PCR are presented. Testing was performed on tenfold serial dilutions of the plasmid containing the 206 bp AIV M gene fragment. (**C**) Clinical sample analysis. Sensitivity comparison of Real-Time LAMP and real-time PCR when testing samples containing various AIV subtypes.

The sensitivity of the LAMP assay using four different dyes was determined by testing tenfold serial dilutions (10^8^ to 10^0^) of a plasmid containing the AIV M gene fragment. All tests were performed in five replicates to ensure reproducibility. The RT-LAMP assay demonstrated detection times of 500–900 s (correlating with high viral loads), while RT-PCR yielded Ct values of 20–35, confirming method validity. Both methods showed 100% concordance in positive sample identification. Real-time LAMP with SYBR Green on the AXXIN system matched real-time PCR results down to a Ct of 38. The assay detected plasmid concentrations from 10^8^ to 10^1^ dilutions (4.9 × 10^1^ copies/μL) with 100% analytical sensitivity (95% CI: 80–100) (Figure 2A,B).

**Figure 3:**
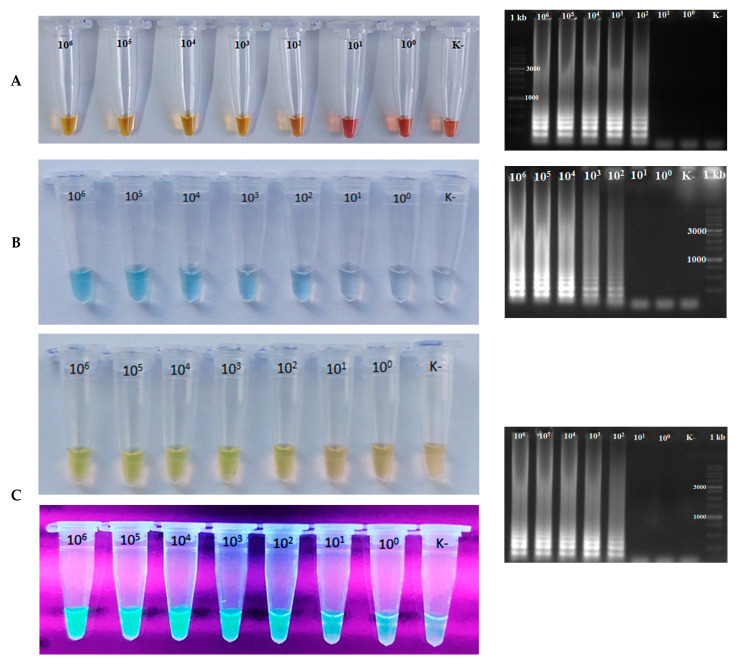
Sensitivity results of optimized LAMP protocols using a plasmid containing a 206-base-pair insert of the M gene fragment of AIV in a series of tenfold dilutions. Data is presented for: (**A**) LAMP test with the colorimetric pH-dependent indicator cresol red and validation of reaction results by agarose gel electrophoresis. (**B**) LAMP test with the colorimetric indicator Malachite Green (0.3% concentration), which changes color during amplification, and validation of reaction results by agarose gel electrophoresis. (**C**) LAMP test with the colorimetric fluorescent indicator calcein (2.5 mM concentration) and validation of reaction results by agarose gel electrophoresis.

Similar tests were performed using cresol red. During the amplification of standard plasmid dilutions by the LAMP method with a colorimetric buffer, changes in pH were observed in samples diluted from 10^6^ to 10^2^ (to 4.9 × 10^2^ copies in µL). In samples diluted from 10^1^ to 10^0^, no pH changes were recorded. These results were confirmed by agarose gel electrophoresis, which did not reveal specific bands in samples diluted from 10^1^ to 10^0^ (Figure 3A).

When assessing the sensitivity of the LAMP method using the colorimetric indicator Malachite Green at a concentration of 0.3%, a color change was observed in samples diluted from 10^6^ to 10^2^ (to 4.9 × 10^2^ copies in µL). No color change was visualized in samples diluted at 10^1^ and 10^0^. Agarose gel electrophoresis confirmed the presence of specific bands in blue-colored samples, verifying positive amplification in the range of dilutions from 10^6^ to 10^2^ (4.9 × 10^6^ to 4.9 × 10^2^ copies in µL) (Figure 3B).

Testing the sensitivity using the fluorescent colorimetric indicator calcein demonstrated similar results to the previously described colorimetric indicators. Fluorescence and a yellow-green color were observed in samples diluted from 10^6^ to 10^2^ (to 4.9 × 10^2^ copies in µL), while no fluorescence and a light brown color were observed in samples diluted from 10^1^ to 10^0^. Agarose gel electrophoresis confirmed the presence of specific bands in positive samples from 10^6^ to 10^2^ (4.9 × 10^6^ to 4.9 × 10^2^ copies in µL) (Figure 3C).

Results obtained using colorimetric LAMP protocols demonstrated lower sensitivity, than real-time PCR and Real-Time LAMP, detecting the plasmid only up to the 10^2^ dilution, which corresponds to the expected sensitivity limit [44] and is comparable to real-time PCR results at Ct = 32 with an analytical sensitivity of 91.67% (95% CI: 76.1–100).

To evaluate the specificity of the optimized LAMP assays for AIV developed in this study, genomic RNA from 34 strains was used, along with a combination of reverse transcription and amplification steps in a single tube. The comparative analysis results demonstrated that Real-Time LAMP with fluorescence detection (Figure 2C), as well as visual formats with cresol red, malachite green, and calcein indicators—exhibited identical and maximally high diagnostic characteristics. All method modifications showed 100% analytical sensitivity (95% CI: 93.7–100) and 100% specificity (95% CI: 82.2–100), which indicates: full compliance with reference methods for positive sample identification, absence of cross-reactions with heterologous viruses and negative control samples and high reproducibility regardless of the method of detections. When testing 27 positive and 7 negative samples, all optimized LAMP formats correctly identified 100% of cases (perfect agreement with reference data) and showed ideal concordance with the gold standard method (Kappa coefficient = 1). The method demonstrates consistently high reproducibility independent of the visualization system used (Table 2).

### 3.3. Results of Field Testing for Optimized LAMP Protocols

During systematic scientific field monitoring of AIV in wild bird populations on the Curonian Spit (Russian Federation) in the spring of 2025, 172 fresh fecal samples from various waterbird species, oropharyngeal and cloacal swabs from 510 captured birds were examined. To assess amplification specificity, various samples of different biological materials from various bird species were selected. For testing optimized LAMP protocols, biological samples were collected from 41 birds, including paired oropharyngeal (*n* = 28) and cloacal (*n* = 28) swabs from 28 bird species (Jack snipe (*Lymnocryptes minimus*), Brambling (*Fringilla montifringilla*), Redwing (*Turdus iliacus*), Dunnock (Prunella modularis), Common firecrest (*Regulus ignicapilla*), Eurasian wren (*Troglodytes troglodytes*), Goldcrest (*Regulus regulus*), Willow warbler (*Phylloscopus trochilus*), European pied flycatcher (*Ficedula hypoleuca*), Common chiffchaff (*Phylloscopus collybita*), Common reed bunting (*Emberiza schoeniclus*), Eurasian chaffinch (*Fringilla coelebs*), European robin (*Erithacus rubecula*), Eurasian blackcap (*Sylvia atricapilla*), Eurasian blue tit (*Cyanistes caeruleus*), Song thrush (*Turdus philomelos*), Common redstart (*Phoenicurus phoenicurus*), Eurasian siskin (*Spinus spinus*), White wagtail (*Motacilla alba*), Hawfinch (*Coccothraustes coccothraustes*), Great tit (*Parus major*), Lesser whitethroat (*Curruca curruca*), Eurasian tree sparrow (*Passer montanus*), European greenfinch (*Chloris chloris*), Common starling (*Sturnus vulgaris*), Black-capped chickadee (*Poecile palustris*), House sparrow (*Passer domesticus*), Great reed warbler (*Acrocephalus arundinaceus*)), as well as 13 fresh fecal samples from European herring gull (*Larus argentatus*, n = 3), Black-headed gull (*Chroicocephalus ridibundus*, n = 2), Gray heron (*Ardea cinerea*, n = 2), Mallard (*Anas platyrhynchos*, n = 2), Great cormorant (*Phalacrocorax carbo*, n=2), and Mute swan (*Cygnus olor*, n = 2). 

All samples underwent a two-stage molecular analysis: initial screening using optimized LAMP protocols with various indicator dyes targeting the AIV M gene, followed by confirmation using real-time PCR.A plasmid containing a 206 bp fragment of the M gene from the avian influenza virus strain A/rook/Russia/A_rook_Nizhniy_Novgorod_2_17/2017(H5N8) at a 10^4^ dilution was used as a positive control to monitor the amplification efficiency of the optimized LAMP protocols during field studies. This control was detected by real-time PCR at a threshold cycle (Ct) value of 25.63.

The results of the scientific field monitoring of AIV in wild bird populations on the Curonian Spit revealed no positive samples. Validation of the optimized LAMP protocols under field conditions demonstrated complete concordance among all diagnostic methods: testing of 69 biological samples (including oropharyngeal and cloacal swabs and fecal samples) collected from 28 passerine species and 6 waterbird species showed consistently negative results with all four LAMP variants (real-time format and visual detection using Cresol Red, Malachite Green, and Calcein dyes) as well as with real-time PCR (with threshold cycle Ct > 40 for all samples). Negative controls showed the expected negative reactions, while positive controls displayed appropriate positive signals, ruling out the possibility of false-negative results and confirming both the absence of avian influenza virus RNA in the tested material and the diagnostic efficacy of the optimized LAMP protocols under field conditions.

## 4. Discussion

Monitoring studies of avian influenza (AI) play a crucial role in understanding the rate of disease spread and organizing quarantine measures in affected areas, particularly for highly pathogenic strains such as H5N1. Early detection through continuous monitoring allows for timely virus identification in both wild and domestic birds, enabling prompt intervention [21,45]. Epidemiological risk assessments conducted as part of surveillance help determine the likelihood of infection transmission, especially in high-risk areas where interactions occur between human, animal, and bird populations [46].

The initiation of quarantine measures is impossible without official confirmation of laboratory test results. During outbreaks on large poultry farms, every minute of inaction can lead to severe and irreversible consequences [47]. The situation regarding timely diagnosis may be exacerbated by the limited number of reference laboratories and subordinate branches capable of conducting necessary molecular genetic analyses [48]. These challenges in performing traditional diagnostic methods, which are time-consuming and require high biosafety levels, further complicate timely diagnosis [49]. In light of these difficulties, the optimal solution could involve the introduction of new AIV diagnostic methods that match the specificity of real-time PCR but do not require adherence to all the restrictions faced by laboratories using this method. One such alternative is LAMP diagnostics, offering a rapid, cost-effective, and user-friendly diagnostic solution—especially important under resource-constrained conditions [41].

Currently, numerous studies have been published on the development of LAMP tests, particularly for diagnosing AIV [41] and its specific subtypes [50,51]. Additionally, methods applicable beyond veterinary use have been described [52]. However, despite the many positive aspects of LAMP tests, authors highlight certain limitations in their development. For example, Jang WS and colleagues noted restricted usability of a multiplex LAMP test for detecting influenza A/B in field conditions due to the need for sterile environments, as all experiments were conducted in a fume hood—not reflective of real-world practical settings. This could lead to cross-contamination via aerosols during manual transfer and mixing of LAMP products with LFA buffer. As a result, consumers may need to purchase additional equipment, such as disposable and affordable pen-like sensors utilizing nucleic acid amplification for at-home infectious disease testing [53].

Despite potential challenges in implementing LAMP tests in field conditions, research in this area continues. For AIV diagnostics, fluorescent indicators such as SYTO 9 and SYBR Green have already been tested for Real-Time LAMP [41,51], as well as commercial colorimetric kits like WarmStart^®^ Colorimetric LAMP 2X Master Mix (DNA & RNA) [54]. However, despite the availability of various colorimetric indicators and published data on their application for different pathogens, they have not yet been tested for AIV diagnostics. It is worth emphasizing that developing various modifications of LAMP tests using highly efficient and specific primers could significantly expand their applications, allowing test systems to be tailored to different user groups based on their needs and usage conditions.

It should be emphasized that the development of various LAMP assay modifications employing highly specific primer systems significantly expands their practical applications, enabling the adaptation of diagnostic platforms to specific user requirements and testing conditions. The successful implementation of the LAMP method largely depends on meticulous primer design, as evidenced by numerous scientific studies. Specifically, Shivakoti et al. (2010) developed a highly sensitive LAMP assay for AIV detection [55], while Yoshida et al. (2011) proposed an optimized primer set (FIP, BIP, LoopF, LoopB) [56]. Of particular interest is the study by Golabi et al. (2021), which analyzed a panel of 800 AIV strains circulating in Europe between 2010 and 2018. The primer system developed by these authors demonstrated exceptional analytical sensitivity (10^2.5^ EID_50_/mL) and rapid detection (15 min), confirmed through experiments with model samples containing AIV. Although the endpoint methodology employed showed resistance to matrix effects at various analyte concentrations, limitations were observed when working with pancreatic and lung tissue samples, manifested through changes in the optical properties of the reaction mixture [41].

In the current study, during optimization of LAMP protocols, the described primer system was successfully applied for detecting conserved regions of the AIV M gene, one more time confirming the high diagnostic efficacy of the optimized LAMP methodologies when used with various colorimetric indicators and suitability for diagnosing various subtypes of AIV. Furthermore, current work demonstrated the feasibility of applying cost-effective and highly efficient protocols for conducting colorimetric LAMP tests in field conditions, underscoring their practical significance. The study also showed comparable efficiency and sensitivity of the Real-Time LAMP method with real-time PCR. Both methods allow for precise and reproducible detection of target nucleic acids. However, Real-Time LAMP required significantly less time compared to traditional methods and was characterized by lower financial costs. Nevertheless, specialized equipment is still necessary for its implementation, although its cost is lower than that of real-time DNA amplifiers. The most cost-effective option is the colorimetric LAMP test, though its sensitivity is lower than that of real-time PCR. When selecting an indicator for visualizing results, it is essential to consider its specific properties. For instance, while calcein allows immediate visualization, interpreting the intensity of color changes may be challenging and prone to errors. For accurate interpretation of results using this indicator, which can be detected both colorimetrically and fluorometrically, specialized equipment is required, such as transilluminators or other UV detection systems. When using cresol red, it is important to note that impurities in samples can affect the reaction pH, potentially leading to false-positive or false-negative results. The protocol using Malachite Green is optimal in terms of speed and cost-effectiveness; however, during hot periods, samples must be cooled for 10 min before visualization. This drawback can easily be addressed with a common coolant, making the method suitable even for field use.

This study confirmed the high diagnostic efficiency of optimized LAMP protocols targeting conserved regions of the AIV M gene. The method demonstrated reliable reproducibility under laboratory conditions and ease of implementation during field monitoring studies, with no occurrence of false-positive results. Comparative analysis revealed that the LAMP method possesses analytical sensitivity comparable to real-time PCR, while offering significant advantages in analysis speed and reduced operational costs. It was established that the colorimetric LAMP modification represents the most economically optimal solution for field diagnostics, despite a slight reduction in sensitivity compared to PCR detection. The primary contribution of this research lies in the development of standardized protocols adapted for use in resource-limited field laboratory settings, highlighting the method’s practical significance for operational epidemiological monitoring of avian influenza directly at sample collection sites.

Thus, the obtained data confirm the high sensitivity and specificity of LAMP tests using various indicators, making them a promising tool for possible primary screening and rapid pathogen detection with minimal laboratory infrastructure. The choice of indicator depends on diagnostic objectives, available equipment, and financial capabilities, highlighting the flexibility and adaptability of the method for various application conditions.

Despite significant advancements in refining LAMP tests, further scientific research is necessary to adapt the method for use in diverse field conditions. It is important to recognize that while monitoring plays a key role in disease control, several issues remain unresolved. Among them is the need to improve data exchange between research and epidemiological centers, as well as adapting surveillance strategies to constantly evolving viral strains.

Continuous innovation and international collaboration are critical to enhancing global preparedness for avian influenza outbreaks. In this context, the development of an effective, affordable, and easy-to-use diagnostic method like LAMP and its adoption as one of the officially approved methods represents a significant step forward. This will not only accelerate pathogen detection but also enhance the timeliness of response to threats, ultimately contributing to improved epidemiological control and reduced risks to birds, animals, and human health.

## 5. Practical Guidelines for AIV Detection Using Optimized LAMP

### 5.1. Target and Validation

Nucleic acids were extracted from clinical samples (oropharyngeal/cloacal swabs or feces) using a commercial magnetic-bead-based kit (Magnoprime Vet). Amplification was performed using optimized LAMP protocols with primers (F3/B3, FIP/BIP, LoopF/LoopB) designed by Golabi et al. (2021) [41], enabling pan-subtype detection of avian influenza virus (AIV), validated on subtypes H1–H12. The assay was calibrated using a 10-fold dilution series of a recombinant plasmid containing a 206 bp fragment of the conserved AIV matrix (M) gene, achieving detection limits of 4.9 × 10^1^ copies/μL for Real-Time RT-LAMP and 4.9 × 10^2^ copies/μL for colorimetric LAMP.

### 5.2. Reaction Setup and Detection

The LAMP reaction was carried out in a 25 μL volume containing: primer mix (FIP/BIP—0.8 μM, LoopF/LoopB—0.4 μM, F3/B3—0.2 μM), 40 U GentaBst DNA polymerase, 50 U MMIv reverse transcriptase (GEN TERRA), and either 5× LAMP buffer (GEN TERRA) or a custom buffer with (NH_4_)_2_SO_4_, MgSO_4_, KCl, Tween-20, and dNTPs. Amplification products were detected using three methods:

Real-Time LAMP: 12.5% SYBR Green (Eurogen) on AXXIN T8 (FAM channel, 58 °C, 30 min);

Cresol red (pH 7.2–8.8): red = negative, yellow = positive (60 °C, 30 min);

Malachite green (0.3%) or calcein (2.5 mM + 25 mM MnCl_2_): blue or green fluorescence = positive (61 °C or 63 °C, 30 min; calcein read under 254 nm UV light).

### 5.3. Result Confirmation

All results were confirmed by real-time RT-PCR (FLI protocol, CFX96, FAM channel, 45 cycles). Amplification products were additionally verified by 1% agarose gel electrophoresis, showing the characteristic ladder-like banding pattern.

### 5.4. Critical Considerations

Perform pre- and post-amplification steps in separate rooms (SanPiN 3.3686-21; MU 1.3.2569-09) to prevent contamination. Avoid acidic/alkaline contaminants with cresol red to prevent false color shifts. Use a UV transilluminator (e.g., VilberLourmat, 254 nm) for calcein—visual interpretation is unreliable. Cool reactions for 10 min before colorimetric reading to stabilize optical properties. Always include positive (M-gene plasmid) and negative (nuclease-free water) controls.

### 5.5. Limitations

Colorimetric LAMP sensitivity: ~10^2^ plasmid copies (Ct ≈ 32 in RT-PCR), lower than Real-Time LAMP (Ct ≈ 38). Does not differentiate AIV subtypes—only confirms presence of influenza A genome. Performance decreases in complex matrices (feces, tissue); RNA purification is essential. Results are qualitative/semi-quantitative only suitable for screening, not quantification.

### 5.6. Sample Preparation and Storage Recommendations

Extract RNA immediately after collection. Short-term storage at +4 °C is acceptable for ≤72 h. For long-term storage, keep samples and RNA at –80 °C; avoid freeze–thaw cycles. Perform LAMP immediately after RNA extraction; if delayed, store RNA at +4 °C (≤24 h) or –80 °C. Do not store at –20 °C. To enhance stability, perform a separate reverse transcription step to generate cDNA, which can be stored at –20 °C.

## 6. Conclusions

Regular monitoring for AIV plays a pivotal role in combating the disease, enabling early outbreak detection and evaluating the effectiveness of control measures. Conducting sample analysis in field conditions is critical for early infection detection and prevents its spread among wild bird populations and poultry farms. This approach ensures timely assessment of the epidemiological situation, aids in identifying new viral strains, and supports targeted control measures, which are vital for maintaining bird health and a favorable epizootic environment.

Currently, one of the most promising alternatives to real-time PCR is the LAMP method, which offers comparable sensitivity and specificity while allowing analysis without complex equipment. The application of LAMP significantly reduces diagnostic time and simplifies the testing process, which is especially important for possible applications in field conditions and situations requiring rapid response to infectious disease outbreaks. Research results in laboratory conditions indicate that the Real-Time LAMP method demonstrates high efficiency comparable to PCR. Meanwhile, the colorimetric LAMP method has slightly lower sensitivity compared to real-time PCR. These findings suggest the potential of colorimetric LAMP as a rapid diagnostic tool for animal diseases. This approach can subsequently be validated in field trials and adapted for diverse conditions to enable primary screening in resource-limited settings where rapid results are essential.

## Figures and Tables

**Table 1:** Pathogens tested using PCR in real time.

№	Pathogen	Ct PCR
1	Avian influenza virus A/chicken/Germany/N/11/49 (H10N7)	26.19
2	Avian influenza virus A/turkey/Wisconsin/66 (H9N2)	24.33
3	Avian influenza virus A/duck/England/56 (H11N6)	22.35
4	Avian influenza virus A/chicken/Pioneer/72 (H9N2)	23.62
5	Avian influenza virus A/turkey/Ontario/6118/68 (H8N4)	25.35
6	Avian influenza virus A/duck/Germany/1215/73 (H2N3)	24.23
7	Avian influenza virus Chekhov/72 (H7N1)	20.07
8	Newcastle disease virus Pestavil	N/A
9	Avian influenza virus A/Uzbekistan (C-68) (H7N2)	25.30
10	Avian influenza virus RU H7	27.85
11	Avian influenza virus A(tern) SA/59 H5N3	26.82
12	Infectious laryngotracheitis virus Bogatichevsky	N/A
13	Infectious laryngotracheitis virus 24a	N/A
14	Avian influenza virus A/chicken/Kaluga/71 (H6N2)	25.65
15	Newcastle disease virus La Sota	N/A
16	Gumboro disease virus 52/70-M	N/A
17	Avian infectious bronchitis virus Connecticut	N/A
18	Avian infectious bronchitis virus Bodett	N/A
19	Avian influenza virus H-52-SPF (H5N3)	24.87
20	Avian influenza virus A/goose/Zavidovo/23/10 (H5N1)	25.26
21	Avian influenza virus A/duck/Novosibirsk/56/05 (H5N1)	20.56
22	Avian influenza virus A/chicken/Sergiev-Posad/1/17 (H5N8)	21.47
23	Avian influenza virus A/rook/Nizhny Novgorod/2/17 (H5N8)	22.62
24	Avian influenza virus A/duck/California/72 (H3N8)	24.68
25	Avian influenza virus A/chicken/USA/PENN/1370/83 (H5N2)	25.74
26	Avian influenza virus A/duck/Czechoslovakia/56 (H4N6)	26.66
27	Avian influenza virus A/duck/Ukraine/1/63 (H3N8)	23.03
28	Avian influenza virus A/seal/Massachusetts/1/80 (H7N7)	21.90
29	Avian influenza virus A/R-42 (H7N1)	20.62
30	Avian influenza virus A/shearwater/Australia/1/72 (H6N5)	22.28
31	Avian influenza virus A/duck/Memphis/546/74 (H11N2)	20.84
32	Avian influenza virus A/black-headed gull/Astrakhan/1421/79 (H13N2)	20.98
33	Avian influenza virus A/gull/Astrakhan/78-4 (H1N1)	26.31
34	Avian influenza virus A/duck/Alberta/60/76 (H12N5)	22.03

**Table 2:** Comparative evaluation of diagnostic performance of optimized LAMP assays with alternative detection indicators versus real-time RT-PCR for AIV and heterologous avian viruses.

Parameters	Real-Time LAMP	LAMP with Cresol Red	LAMP with Malachite Green	LAMP with Calcein
Analytical Sensitivity	100% (95% CI: 93.7–100)	100% (95% CI: 93.7–100)	100% (95% CI: 93.7–100)	100% (95% CI: 93.7–100)
Analytical Specificity	100%(95% CI: 82.2–100)	100%(95% CI: 82.2–100)	100%(95% CI: 82.2–100)	100%(95% CI: 82.2–100)
Test Results				
- Positive results (reference)	27	27	27	27
- Positive results (test)	27	27	27	27
- Negative results (reference)	7	7	7	7
- Negative results (test)	7	7	7	7
Kappa coefficient	1	1	1	1

## Data Availability

The original contributions presented in this study are included in the article/Supplementary Material. Further inquiries can be directed to the corresponding authors.

## Supplementary Materials

The following supporting information can be downloaded at: https://www.mdpi.com/article/10.3390/ani15202983/s1, Table S1 (Composition of the reaction mixture for Real-Time LAMP with SYBR Green indicator), Table S2 (Composition of the reaction mixture for Colorimetric LAMP with malachite green indicator), Table S3 (Composition of the reaction mixture for Colorimetric LAMP with calcein indicator), Table S4 (Composition of the reaction mixture for Colorimetric LAMP with Cresol Red indicator), Table S5 (Composition of the Reaction Buffer for Colorimetric LAMP with Cresol Red Indicator).

## References

[1] Paukett M., Dharmasena S., Bessler D.A. (2018). Revenue impacts of the 2015 avian influenza virus outbreak on the United States table egg wholesalers. Soc. Sci. Res. Netw..

[2] Palù G., Roggero P.F., Calistri A. (2024). Could H5N1 bird flu virus be the cause of the next human pandemic?. Front. Microbiol..

[3] Nicita A. (2008). Avian influenza and the poultry trade. Policy Research Working Paper Series.

[4] Narrod C., Otter J., Pfeiffer D. (2007). Using risk analysis to capture the spatial spread of avian influenza, evaluating the cost-effectiveness of alternative control strategies, and assessing the impact on the poor. Environmental Informatics and Systems Research.

[5] Hagerman A.D., Marsh T.L. (2016). Theme overview: Economic consequences of highly pathogenic avian influenza. Choices Mag. Food Farm Resour. Issues.

[6] Reuters (2025). UK Detects Human Case of Bird Flu, Says Wider Risk Remains Low. https://www.reuters.com/business/healthcare-pharmaceuticals/uk-detects-human-case-bird-flu-2025-01-27.

[7] Pekarek M.J., Weaver E.A. (2024). Influenza B virus vaccine innovation through computational design. Pathogens.

[8] Kaverin N.V., Lvov D.K. (2008). Orthomyxoviruses (Orthomyxoviridae). Medical Virology.

[9] Air G.M., Compans R.W. (1983). Influenza B and influenza C viruses. Springer eBooks.

[10] Hause B.M., Collin E.A., Liu R., Huang B., Sheng Z., Lu W., Wang D., A Nelson E., Li F. (2014). Characterization of a novel influenza virus in cattle and swine: Proposal for a new genus in the Orthomyxoviridae family. mBio.

[11] Kolobukhina L.V., Lvov D.K., Burtseva E.I. (2008). Influenza. Medical Virology.

[12] Khantimirova L.M., Guseva S.G., Shevtsov V.A., Merkulov V.A., Bondarev V.P. (2020). Comparative analysis of approaches to assess the quality of inactivated influenza vaccines: Regulatory requirements in the Russian Federation and European Union. Epidemiol. Vaccinal Prev..

[13] Shen J., Zhang H., Sun X., Zhang Y., Wang M., Guan M., Liu L., Li W., Xu H., Xie Y. (2024). Evolution and biological characteristics of H11 avian influenza viruses isolated from migratory birds and pigeons. Emerg. Microbes Infect..

[14] Lv X., Tian J., Li X., Bai X., Li Y., Li M., An Q., Song X., Xu Y., Sun H. (2023). H10Nx avian influenza viruses detected in wild birds in China pose potential threat to mammals. One Health.

[15] Yoon S.W., Webby R.J., Webster R.G. (2014). Evolution and ecology of influenza A viruses. Curr. Top. Microbiol. Immunol..

[16] Murashkina T., Sharshov K., Gadzhiev A., Petherbridge G., Derko A., Sobolev I., Dubovitskiy N., Loginova A., Kurskaya O., Kasianov N. (2024). Avian influenza virus and avian paramyxoviruses in wild waterfowl of the Western Coast of the Caspian Sea (2017–2020). Viruses.

[17] Blagodatski A., Trutneva K., Glazova O., Mityaeva O., Shevkova L., Kegeles E., Onyanov N., Fede K., Maznina A., Khavina E. (2021). Avian influenza in wild birds and poultry: Dissemination pathways, monitoring methods, and virus ecology. Pathogens.

[18] Bravo-Vasquez N., Schultz-Cherry S. (2021). Avian influenza viruses. Encyclopedia of Virology.

[19] World Organisation for Animal Health (WOAH) WAHIS Dashboard 2025. https://wahis.woah.org/#/dashboards/qd-dashboard.

[20] Alexakis L., Buczkowski H., Ducatez M., Fusaro A., Gonzales J.L., Kuiken T., Ståhl K., Staubach C., Svartström O., Terregino C. (2024). Avian influenza overview June–September 2024. EFSA J..

[21] Wang W., Sun Y., Li Z., Yang W., Feng S. (2024). Are we prepared for the next pandemic: Monitor on increasing human and animal H5N1 avian influenza infection. Chin. Med. J..

[22] World Organisation for Animal Health (WOAH) (2025). Avian Influenza (Terrestrial Animal Health Code). https://www.woah.org/fileadmin/Home/eng/Health_standards/tahm/3.03.04_AI.pdf.

[23] Moure Z., Cuadros E., Pablo-Marcos D., Reina M.J., de Benito I., Campo A.B. (2023). Establishing a molecular laboratory in COVID-19 pandemic: The experience of a regional laboratory in Spain. Acta Microbiol. et Immunol. Hung..

[24] Sathyanarayana S.H., Wainman L.M. (2024). Laboratory approaches in molecular pathology: The polymerase chain reaction. Diagnostic Molecular Pathology.

[25] Huang R. (2023). Guidance for molecular diagnostic laboratory. Diagnostic Molecular Biology.

[26] Srivastava P., Prasad D. (2023). Isothermal nucleic acid amplification and its uses in modern diagnostic technologies. 3 Biotech.

[27] Zhou S. (2022). Rapid and highly specific detection of communicable pathogens using one-pot loop probe-mediated isothermal amplification (oLAMP). Sens. Actuators B Chem..

[28] Wongsawat K., Dharakul T., Narat P., Rabablert J. (2011). Detection of nucleic acid of classical swine fever virus by reverse transcription loop-mediated isothermal amplification (RT-LAMP). Health.

[29] Kim M., Shin S.W., Shin J., Kim E., Yang S.-M., Gwak Y.-S., Lee S., Kim H.-Y. (2024). Loop-mediated isothermal amplification (LAMP) assays for rapid on-site detection of *Lacticaseibacillus casei*, *Lacticaseibacillus paracasei*, and *Lacticaseibacillus rhamnosus* in probiotic products. Food Biosci..

[30] LaPointe V.L.S., Roy M., Rose S.L., Boutin Y., Couture F. (2024). Conception and optimization of extraction-free loop-mediated isothermal amplification detection of dry rot fungus *Serpula lacrymans*. ACS Omega.

[31] Yang L., Wang L., Lv M., Sun Y., Cao J. (2022). Clinical validation of DNA extraction-free qPCR, visual LAMP, and fluorescent LAMP assays for the rapid detection of African swine fever virus. Life.

[32] Kang J., Seo M.-R., Chung Y.-J. (2022). Development of reverse-transcription loop-mediated isothermal amplification assays for point-of-care testing of human influenza virus subtypes H1N1 and H3N2. Genom. Inform..

[33] Simon D.S., Yew C.W., Kumar V.S. (2023). Multiplexed reverse transcription loop-mediated isothermal amplification coupled with a nucleic acid-based lateral flow dipstick as a rapid diagnostic method to detect SARS-CoV-2. Microorganisms.

[34] Sahni A.K., Grover N., Sharma A., Khan I.D., Kishore J. (2013). Reverse transcription loop-mediated isothermal amplification (RT-LAMP) for diagnosis of dengue. Med. J. Armed Forces India.

[35] Parida M., Posadas G., Inoue S., Hasebe F., Morita K. (2004). Real-time reverse transcription loop-mediated isothermal amplification for rapid detection of West Nile virus. J. Clin. Microbiol..

[36] PHARMA (2025). Gene-8C Isothermal Fluorescent PCR Amplifier. https://pharma-se.ru/equipments/biology/ptsr-amplifikatory/izotermicheskiy-fluorestsentnyy-ptsr-amplifikator-gene-8c/?set_filter=y.

[37] AXXIN (2025). T8 Isothermal Amplifier. https://www.axxin.com/t8-iso.

[38] PCRBOT (2025). Portable PCR Devices. https://pcrbot.ru/.

[39] Ministry of Health of the Russian Federation (2021). On the Approval of Sanitary Rules and Regulations SanPiN 3.3686-21.

[40] State Sanitation and Epidemiological Regulation of the Russian Federation (2009). Methodological Guidelines (MU 1.3.2569-09).

[41] Golabi M., Flodrops M., Grasland B., Vinayaka A.C., Quyen T.L., Nguyen T., Bang D.D., Wolff A. (2021). Development of reverse transcription loop-mediated isothermal amplification assay for rapid and on-site detection of avian influenza virus. Front. Cell. Infect. Microbiol..

[42] Navarro F., Martínez F.F. (2022). Colorimetric LAMP/RT-LAMP Protocol. Protocols.io.

[43] Spackman E., Senne D.A., Myers T.J., Bulaga L.L., Garber L.P., Perdue M.L., Lohman K., Daum L.T., Suarez D.L. (2002). Development of a real-time reverse transcriptase PCR assay for type A influenza virus and the avian H5 and H7 hemagglutinin subtypes. J. Clin. Microbiol..

[44] Werbajh S., Larocca L., Carrillo C., Stolowicz F., Ogas L., Pallotto S., Cassará S., Mammana L., Zapiola I., Bouzas M.B. (2023). Colorimetric RT-LAMP detection of multiple SARS-CoV-2 variants and lineages of concern direct from nasopharyngeal swab samples without RNA isolation. Viruses.

[45] Duan C., Li C., Ren R., Bai W., Zhou L. (2023). An overview of avian influenza surveillance strategies and modes. Sci. One Health.

[46] Schoene C., Staubach C., Grund C., Globig A., Kramer M., Wilking H., Beer M., Conraths F.J., Harder T.C., the BL Monitoring Group (2013). Towards a new, ecologically targeted approach to monitoring wild bird populations for avian influenza viruses. Epidemiol. Infect..

[47] Slomka M.J., To T.L., Tong H.H., Coward V.J., Hanna A., Shell W., Pavlidis T., Densham A.L.E., Kargiolakis G., Arnold M.E. (2012). Challenges for accurate and prompt molecular diagnosis of clades of highly pathogenic avian influenza H5N1 viruses emerging in Vietnam. Avian Pathol..

[48] Rai M., Mishra A. (2018). Diagnosis of AIV by molecular diagnostic techniques. Int. J. Chem. Stud..

[49] Haryanto A., Krisanti B., Irianingsih S.H., Yudianingtyas D.W. (2013). Molecular diagnosis of avian influenza virus type A and subtype H5 by amplification of its M and H5 genes using one step simplex RT-PCR. J. Vet..

[50] Fan Q., Xie Z., Zhao J., Hua J., Wei Y., Li X., Li D., Luo S., Li M., Xie L. (2024). Simultaneous differential detection of H5, H7, and H9 subtypes of avian influenza viruses by a triplex fluorescence loop-mediated isothermal amplification assay. Front. Vet. Sci..

[51] Zhang S., Shin J., Shin S., Chung Y.J. (2020). Development of reverse transcription loop-mediated isothermal amplification assays for point-of-care testing of avian influenza virus subtype H5 and H9. Genom. Inform..

[52] Jang W.S., Lee J.M., Lee E., Park S., Lim C.S. (2024). Loop-mediated isothermal amplification and lateral flow immunochromatography technology for rapid diagnosis of influenza A/B. Diagnostics.

[53] Lu X., Lin H., Feng X., Lui G.C.Y., Hsing I.-M. (2022). Disposable and low-cost pen-like sensor incorporating nucleic-acid amplification based lateral-flow assay for at-home tests of communicable pathogens. Biosens. Bioelectron..

[54] Ahn S.J., Baek Y.H., Lloren K.K.S., Choi W.-S., Jeong J.H., Antigua K.J.C., Kwon H.-I., Park S.-J., Kim E.-H., Kim Y.-I. (2019). Rapid and simple colorimetric detection of multiple influenza viruses infecting humans using a reverse transcriptional loop-mediated isothermal amplification (RT-LAMP) diagnostic platform. BMC Infect. Dis..

[55] Shivakoti S., Ito H., Murase T., Ono E., Takakuwa H., Yamashiro T., Otsuki K., Ito T. (2010). Development of reverse transcription-loop-mediated isothermal amplification (RT-LAMP) assay for detection of avian influenza viruses in field specimens. J. Vet. Med. Sci..

[56] Yoshida H., Sakoda Y., Endo M., Motoshima M., Yoshino F., Yamamoto N., Okamatsu M., Soejima T., Senba S., Kanda H. (2011). Evaluation of the reverse transcription loop-mediated isothermal amplification (RT-LAMP) as a screening method for the detection of influenza viruses in the fecal materials of water birds. J. Vet. Med. Sci..

