# Supplementary material for: Optimization of Loop-Mediated Isothermal Amplification for Avian Influenza Detection
Source: Animals (Basel). 2025 Oct 15;15(20):2983. doi: 10.3390/ani15202983 (PMC12560903; doi:10.3390/ani15202983)
Supplement: Supplementary file 1 [file animals-15-02983-s001.zip › animals-3868032-supplementary.pdf]

Table S1. Composition of the reaction mixture for Real-Time LAMP with SYBR Green indicator.

Component	Volume per reaction	Description
20 μM, F3/B3, FIP/BIP, LF/LB	1,75 μL	Mixture of published primers at working concentration, Eurogen (Russia)
5x Genta LAMP buffer	5 μL	Buffer for providing optimal reaction conditions, GENTERRA (Russia)
10 mM dNTP	3,5 μL	Deoxynucleotide triphosphates (dATP, dGTP, dCTP, dTTP) in TE buffer, diaGene (Russia)
50X SYBR Green I (12,5X)	0,6 μL	Fluorescent dye for amplification detection, Evrogen (Russia)
DNA polymerase Genta Bst (8000 U/mL)	1 μL	DNA polymerase Genta Bst, GENTERRA (Russia)
Deionized water	Up to 25 μL	Nuclease-free water, Eurogen (Russia)

Table S2. Composition of the reaction mixture for Colorimetric LAMP with malachite green indicator.

Component	Volume per reaction	Description
20 μM, F3/B3, FIP/BIP, LF/LB	1,75 μL	Mixture of published primers at working concentration, Eurogen (Russia)
5x Genta LAMP buffer	5 μL	Buffer for providing optimal reaction conditions, GENTERRA (Russia)
25 mM Dntp (10 mM)	3,5 μL	Deoxynucleotide triphosphates (dATP, dGTP, dCTP, dTTP) in TE buffer, diaGene (Russia)
1% Malachite Green (0,3%)	1 μL	Colorimetric indicator, HiMedia (India)
DNA polymerase Genta Bst (8000 U/mL)	1 μL	DNA polymerase Genta Bst, GENTERRA (Russia)
Deionized water	Up to 25 μL	Nuclease-free water, Eurogen (Russia)

Table S3. Composition of the reaction mixture for Colorimetric LAMP with calcein indicator.

Component	Volume per reaction	Description
20 μM, F3/B3, FIP/BIP, LF/LB	1,75 μL	Mixture of published primers at working concentration, Eurogen (Russia)
5x Genta LAMP buffer	5 μL	Buffer for providing optimal reaction conditions, GENTERRA (Russia)
25 mM Dntp (10 mM)	3,5 μL	Deoxynucleotide triphosphates

		(dATP, dGTP, dCTP, dTTP) in TE buffer, diaGene (Russia)
25 mM $MnCl_2$	0,67 µL	
2,5 mM Calcein	0,33 µL	Fluorescein complexone AR, CDH, (India)
DNA polymerase Genta Bst (8000 U/mL)	1 µL	DNA polymerase Genta Bst, GENTERRA (Russia)
Deionized water	До 25 µL	Nuclease-free water, Eurogen (Russia)

Table S4. Composition of the reaction mixture for Colorimetric LAMP with Cresol Red indicator.

Component	Volume per reaction	Description
20 µM, F3/B3, FIP/BIP, LF/LB	1,75 µL	Mixture of published primers at working concentration, Eurogen (Russia)
LAMP buffer	12,5 µL	Buffer for providing optimal reaction conditions, GENTERRA (Russia)
DNA polymerase Genta Bst (8000 U/mL)	1 µL	DNA polymerase Genta Bst, GENTERRA (Russia)
Deionized water	Up to 25 µL	Nuclease-free water, Eurogen (Russia)

Table S5. Composition of the Reaction Buffer for Colorimetric LAMP with Cresol Red Indicator

Reagents	Stock Solution Concentrations	Buffer Solution	Volume
dNTPs	10 mM	2.8 mM	280 µL
$(NH_4)_2SO_4$	1 M	20 mM	20 µL
$MgSO_4$	1 M	16 mM	16 µL
KCl	1 M	100 mM	100 µL
Tween 20	100%	0.2%	2 µL
Cresol Red	1 M	1.25 mM	125 µL
Total volume of mixture			1000 µL
